# Carbohydrate restriction following strenuous glycogen-depleting exercise does not potentiate the acute molecular response associated with mitochondrial biogenesis in human skeletal muscle

**DOI:** 10.1007/s00421-021-04594-8

**Published:** 2021-02-10

**Authors:** Catarina Ramos, Arthur J. Cheng, Sigitas Kamandulis, Andrejus Subocius, Marius Brazaitis, Tomas Venckunas, Thomas Chaillou

**Affiliations:** 1 School of Health Sciences, Örebro University, 701 82 Örebro, Sweden; 2 Department of Physiology and Pharmacology, Karolinska Institutet, 171 77 Stockholm, Sweden; 3 Muscle Health Research Centre, School of Kinesiology and Health Sciences, Faculty of Health, York University, Toronto, M3J 1P3 Canada; 4 Sports Science and Innovation Institute, Lithuanian Sports University, 44221 Kaunas, Lithuania; 5 Department of Surgery, Kaunas Clinical Hospital, 47144 Kaunas, Lithuania; 6 Clinic of Surgery, Republican Hospital of Kaunas, 45130 Kaunas, Lithuania

**Keywords:** Oxidative metabolism, Train-low, Muscle glycogen, PGC1A, Sprint interval exercise, Endurance exercise

## Abstract

**Purpose:**

Carbohydrate (CHO) restriction could be a potent metabolic regulator of endurance exercise-induced muscle adaptations. Here, we determined whether post-exercise CHO restriction following strenuous exercise combining continuous cycling exercise (CCE) and sprint interval exercise could affect the gene expression related to mitochondrial biogenesis and oxidative metabolism in human skeletal muscle.

**Methods:**

In a randomized cross-over design, 8 recreationally active males performed two cycling exercise sessions separated by 4 weeks. Each session consisted of 60-min CCE and six 30-s all-out sprints, which was followed by ingestion of either a CHO or placebo beverage in the post-exercise recovery period. Muscle glycogen concentration and the mRNA levels of several genes related to mitochondrial biogenesis and oxidative metabolism were determined before, immediately after, and at 3 h after exercise.

**Results:**

Compared to pre-exercise, strenuous cycling led to a severe muscle glycogen depletion (> 90%) and induced a large increase in *PGC1A* and *PDK4* mRNA levels (~ 20-fold and ~ 10-fold, respectively) during the acute recovery period in both trials. The abundance of the other transcripts was not changed or was only moderately increased during this period. CHO restriction during the 3-h post-exercise period blunted muscle glycogen resynthesis but did not increase the mRNA levels of genes associated with muscle adaptation to endurance exercise, as compared with abundant post-exercise CHO consumption.

**Conclusion:**

CHO restriction after a glycogen-depleting and metabolically-demanding cycling session is not effective for increasing the acute mRNA levels of genes involved in mitochondrial biogenesis and oxidative metabolism in human skeletal muscle.

## Introduction

Carbohydrate (CHO) is a crucial macronutrient used to fuel skeletal muscle during exercise and restore glycogen after exercise (Bergstrom et al. 1967). Muscle glycogen, which is the major source of endogenous CHO, is highly depleted following prolonged and exhausting exercise (Cheng et al. 2020; Greiwe et al. 1999), and enhancing its resynthesis with CHO supplementation can accelerate recovery (Bergstrom et al. 1967; Alghannam et al. 2016). In contrast, CHO restriction has been proposed to be a potential regulator of intracellular signaling pathways promoting endurance-training adaptations in skeletal muscle (e.g., mitochondrial biogenesis, substrate utilization, oxidative metabolism) (Hearris et al. 2018; Mata et al. 2019).

Over the last two decades, the “train-low” concept that consists of purposely reducing glycogen stores and/or restricting CHO intake to enhance training adaptations has been widely used by endurance athletes (Bartlett et al. 2015; Hearris et al. 2018). Some evidence indicates that a reduction of pre-exercise CHO availability (due to an initial glycogen-depleting exercise combined with restricted CHO intake) potentiates the exercise-induced activation of signaling pathways associated with endurance-training adaptations in skeletal muscle (Psilander et al. 2013; Yeo et al. 2010; Bartlett et al. 2013). One critical limitation of reducing pre-exercise CHO availability is the decline of physical capacity during a subsequent intense exercise session performed until exhaustion (Hearris et al. 2019). Thus, this strategy is not optimal during specific periodization phases when endurance athletes need to perform strenuous exercises at high intensities several times per week.

One option to avoid the reduction of physical capacity during intense training sessions while maximizing the acute molecular response to endurance exercise could be to subject the athletes to post-exercise CHO restriction for a few hours directly after a strenuous endurance exercise. With such an approach, muscle glycogen stores could be restored within 24 h if an appropriate CHO-enriched diet is ingested following the acute CHO-restricted period (Jensen et al. 2015), allowing the athletes to be physically recovered for the subsequent intense workout. Previous studies indicated that CHO restriction following prolonged continuous exercise that induces moderate muscle glycogen-depleting (~ 40–65%) does not affect the mRNA levels of genes involved in mitochondrial biogenesis and muscle metabolism during the acute 2–5 h post-exercise recovery period (Jensen et al. 2015; Pilegaard et al. 2005). It was recently proposed that the acute increased expression of exercise-responsive genes and the chronic training adaptations associated with “train-low” protocols could be optimal when muscle glycogen depletion is severe (Impey et al. 2018; Hammond et al. 2019). Despite the pronounced muscle glycogen depletion (~ 75%) directly observed after an exhaustive cycling session performed at moderate intensity, the expression of key regulators of muscle adaptation was not affected by low CHO intake during the recovery period (Mathai et al. 2008). Thus, acute CHO restriction after prolonged and moderate-intensity exercise that induced severe muscle glycogen depletion does not seem to increase the acute expression (2–5 h post exercise) of genes related to mitochondrial biogenesis and oxidative metabolism.

Peroxisome proliferator-activated γ-receptor coactivator 1α (PGC-1α) is a transcriptional coactivator that governs the expression of numerous genes involved in endurance exercise-induced muscle adaptation, thus orchestrating key processes, such as mitochondrial biogenesis and oxidative metabolism (Correia et al. 2015). Prolonged moderate-intensity continuous cycling exercises (CCE) induced an increase in *PGC1A* mRNA levels, a response that was not influenced by post-exercise CHO restriction during the early recovery phase (2–5 h) (Pilegaard et al. 2005; Jensen et al. 2015; Mathai et al. 2008). However, high mRNA levels of *PGC1A* were still observed when post-exercise CHO restriction was maintained for a longer period (i.e., 8 h) (Pilegaard et al. 2005). Furthermore, sprint interval exercise (SIE), which consists of brief supramaximal efforts interspaced with resting periods, is a highly metabolically-demanding exercise that also greatly increases *PGC1A* mRNA levels (Cochran et al. 2014). Therefore, it is conceivable that performing a strenuous exercise session consisting of CCE followed by SIE, and which results in a drastic muscle glycogen depletion (Cheng et al. 2020), could answer the question as to whether post-exercise CHO restriction enhances the acute expression of *PGC1A* and other mitochondrial biogenesis-related genes. In this context where both severe muscle glycogen depletion and high metabolic stress are induced, we hypothesized that post-exercise CHO restriction may be beneficial to potentiate the acute molecular response associated with stimulation of mitochondrial biogenesis and oxidative metabolism. To test this hypothesis, we determined the effect of post-exercise CHO restriction following an exercise session combining CCE and SIE on the acute gene expression related to exercise-induced muscle adaptations in humans.

## Materials and methods

This work is a follow-up to a recently published study (Cheng et al. 2020). The information related to the experimental design, the exercise sessions, CHO intake and nutritional control has already been described in the previous article. In this latter study, we determined whether CHO ingestion could accelerate recovery of prolonged low-frequency force depression induced by a glycogen-depleting exercise session combining moderate-intensity and high-intensity sprint exercises.

### Participants

As previously described (Cheng et al. 2020), a randomized crossover design was used, in which eleven healthy male participants initially volunteered to participate. The inclusion criteria used in the current study were: aged between 18 and 40 years, being healthy and physically active with normal blood pressure and without medication. The exclusion criteria were: suffering from any kind of disease, having an injury or any other conditions that would compromise the ability to perform the physical tests. Due to the difficulty of recruiting endurance athletes, we only included recreationally active males who regularly exercised up to 5 h/week. It is noteworthy that one participant withdrew during the study and two subjects needed to be excluded from the muscle biological analysis (due to low amount of biological material available or technical issues during RNA isolation). Therefore, eight participants [age: 32.4 ± 6.4 years; height: 185.8 ± 4.6 cm; body mass: 86.8 ± 9.9 kg; percentage body fat: 16.6 ± 5.0%; body mass index: 25.2 ± 3.2 kg/m^2^; *V*O_2max_: 49.9 ± 8.9 mL/kg/min] were finally included in this study. A similar sample size (i.e., *N* = 7–9) was used in previous studies that analyzed the effects on CHO restriction following exercise on the mRNA levels of genes involved in endurance-training adaptations in skeletal muscle (Pilegaard et al. 2005; Jensen et al. 2015; Mathai et al. 2008). The percentage body fat was measured using bio-electrical impedance analysis (Tanita TBF-300 UK Ltd, West Drayton, UK) and *V*O_2max_ was determined as previously described (Cheng et al. 2020). The distribution of myosin heavy chain (MHC) isoforms (as described below) was: MHC1: 36.9 ± 13.6%; MHC2A: 54.2 ± 9.4%; MHC2X: 8.9 ± 8.6%. This study was approved by the Kaunas Regional Research Ethics Committee (no. BE-2-17) and was performed in accordance with the last revision of the Declaration of Helsinki.

### Experimental design and exercise session

The experimental design was previously described (Cheng et al. 2020). Briefly, the experiments consisted of three visits to the laboratory. During the first visit, a maximal incremental cycling test was performed to determine *V*O_2max_ and the intensity used during the subsequent prolonged moderate-intensity cycling exercise executed in visits 2–3. During this first visit, the participants were also familiarized with the equipment and protocols used during the subsequent two visits. The second visit was performed approximately a week after the first visit. The two experimental trials (visits 2–3) were completed in a random order and were separated by four weeks during which the participants maintained their regular physical activity. The participants were asked to refrain from any strenuous exercise during the last 3 days prior to each experimental session.

**Fig. 1:**
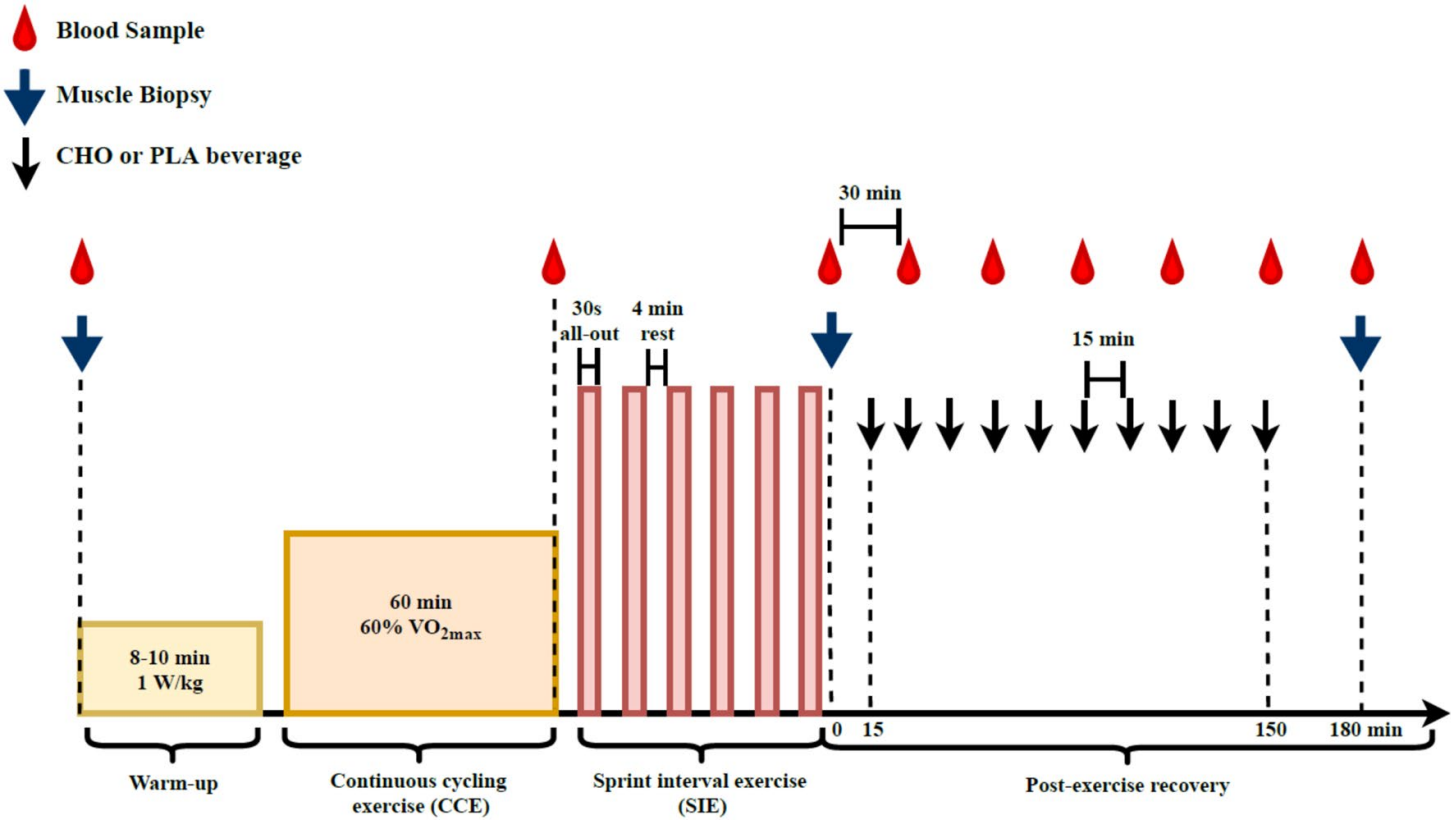
Overview of the experimental protocol performed during the two randomized trials. After a warm-up at low intensity (yellow bar), the participants performed a 60-min continuous cycling exercise (orange bar) followed by six 30-s all-out cycling sprints (light pink bar). From 15 to 150 min post-exercise, the subjects were given a carbohydrate (CHO) or a placebo (PLA) beverage. The neuromuscular testing performed and analyzed in the initial study (Cheng et al. 2020) is not included in this figure. This figure was adapted from Cheng et al. (2020)

In visits 2–3, the participants arrived at the laboratory in the morning after a 10–12 h overnight fast and an initial biopsy was collected from the vastus lateralis muscle. An overview of the experimental protocol is presented in Fig. 1. It is noteworthy that neuromuscular testing of knee extensors (i.e. electrically evoked torque and maximal voluntary contraction) was executed before (1 test) and after exercise (7 tests). These tests were only performed on the dominant leg, and thus did not interfere with the muscle biological analyses since muscle biopsies were collected from the vastus lateralis muscle of the non-dominant leg (see below). These tests, performed to answer the aim of the initial study (Cheng et al. 2020), are not included in the current analysis. After muscle biopsy, a warm-up was performed, consisting of 8–10 min of cycling at 1 W/kg body mass, followed by light active stretching of the knee extensor muscles and by 2–3 short isometric knee extensions in the dynamometer chair. This latter exercise was used as a warm-up for the neuromuscular testing. After the completion of the baseline neuromuscular testing (~ 10 min), the participants performed a cycling session involving CCE followed by SIE. For a full description of the exercise session, refer to Cheng et al. (2020). In short, this session consisted of performing a 60-min continuous cycling exercise (i.e. CCE) at a power eliciting 60% *V*O_2max_ followed by six 30-s all-out cycling sprints (SIE) interspaced with 4 min recovery. The 60-min CCE was performed on an electromechanically braked cycling ergometer (Ergoselect 200P, Ergoline, Medical Measurement Systems, Binz, Germany), and a short break (2–3 min) was allowed after 30 min of CCE during which the participants could drink water (up to 0.5 L). Heart rate (HR) and the rating of perceived exertion (RPE, 6–20 scale) were determined every 10 min during the CCE to evaluate the perceived effort level. The all-out cycling sprints were performed using a mechanically braked cycling ergometer (Monark 824E, Monark, Vansbro, Sweden) with a brake weight corresponding to 7.5% of the participant’s body mass. The peak HR of each cycling sprint and average power were obtained during the SIE. A second muscle biopsy was collected 2–3 min after the last cycling sprint. In a random order, the participants ingested a beverage containing either carbohydrate (CHO) or placebo (PLA) every 15 min during the 150-min period following the completion of the last sprint (post-exercise recovery period). A third biopsy was collected 180 min after the last sprint (i.e. 30 min after the last beverage ingestion). Capillary blood concentration of glucose and lactate was determined from the fingertip before exercise, immediately after CCE, immediately after SIE, and every 30 min during the 180-min recovery period, as previously described (Cheng et al. 2020). Additional neuromuscular testing was executed 15, 30, 60, 90, 120, 150 and 180 min after the completion of the last cycling sprint (not included in the analysis).

### Carbohydrate intake during the recovery period and nutritional control

The CHO-enriched beverage contained glucose and fructose at a 2:1 ratio. CHO (final concentration: 100 g/L) was dissolved in a solution containing water and 1:20 lemon juice concentrate (22 g carbohydrate/L of concentrate; Solevita). The volume of CHO beverage was adjusted for each participant to provide 1.5 g/kg body mass/h CHO during the recovery period. An equal volume was ingested every 15 min following the last cycling sprint over a 150-min period to provide the adequate amount of CHO. The total amount of CHO ingested during the CHO trial (i.e. 150-min post-exercise period) was 328.9 ± 37.5 g. The rationale behind this nutritional approach was previously described (Cheng et al. 2020). The PLA beverage was prepared using a solution of water with lemon juice concentrate (1:20 dilution), in which 0.6 g/L of sodium saccharin (Hermesetas, Zurich, Switzerland) and 4 g/L of Suketter (containing sodium cyclamate and sodium saccharin; Cederroth, Upplands Väsby, Sweden) were dissolved. The total amount of CHO ingested during the PLA trial (i.e. 150-min post-exercise period) was negligible (3.6 ± 0.4 g). The volume of beverage consumed was identical for both CHO and PLA trials.

As described above, the participants performed the strenuous exercise sessions in the morning after a 10–12 h overnight fast. They were asked to take a similar meal on the evening before each exercise session and to refrain from any food and caffeine intakes on the morning before each exercise session. Overnight fasting was chosen for two main reasons: (1) to reduce the risks of gastrointestinal issues and vomiting commonly observed during or immediately after all-out cycling sprints; (2) to restrict CHO (that would have been available in the blood stream after breakfast) during exercise, thereby promoting large muscle glycogen depletion. A similar strategy (strenuous exercise after overnight fasting) was accompanied by very low muscle glycogen concentrations after exercise in untrained and trained participants (Hickner et al. 1997; Greiwe et al. 1999). It is also noteworthy that pre-exercise muscle glycogen concentration (see in the result section) was in the same range as that observed in the literature after overnight fast in a similar population (Blom et al. 1987; Maehlum et al. 1977).

### Muscle biopsies

Needle biopsies from the non-dominant vastus lateralis muscle were collected by a trained physician, as previously described (Cheng et al. 2020). The non-dominant leg was not subjected to neuromuscular testing. Three biopsy samples (~ 10 mg each) were taken before exercise, 2–3 min after and 180 min after the last cycling sprint. The samples were immediately frozen in liquid nitrogen and stored at − 80 °C until further analysis.

### Muscle glycogen measurements

Glycogen concentration was assessed using 5–10 mg of frozen muscle, as previously described (Cheng et al. 2020). Briefly, muscle samples were dissolved in 25 volumes of 2 M NaOH for 50 min at 95 °C, before being neutralized with an equal volume of 2 M HCl. The homogenates (1:50 dilution) were first diluted with distilled water (final dilution: 1:400), and then 5 µL of each diluted homogenate was loaded in duplicates into a 96-well plate. The samples were then analyzed using a fluorometric kit (ab65620, Abcam, Cambridge, UK) following the manufacturer’s instructions. Due to technical reasons, muscle glycogen concentrations could not be expressed in mmol glucosyl units/kg dry weight and were instead expressed as mmol glucosyl units/kg wet weight.

### Distribution of myosin heavy chain (MHC) isoforms

The distribution of MHC isoforms was assessed using 5–10 mg of frozen muscle collected before exercise. Muscle samples were homogenized with a bead-homogenizer (TissueLyser LT, Qiagen, Sollentuna, Sweden) in ice-cold lysis buffer (20 µL/mg; pH 7.6) containing 20 mM Hepes, 150 mM NaCl, 5 mM EDTA, 25 mM KF, 1 mM Na_3_VO_4_, 5% glycerol, 0.5% Triton X-100, and EDTA-free protease inhibitor cocktail (1 tablet/10 mL buffer; # 04 693 159 001, Sigma-Aldrich, Stockholm, Sweden). Protein concentration of the homogenates was determined using BCA protein assay (#23227, Thermo Fisher Scientific, Stockholm, Sweden), and then adjusted to 2 µg/µL after dilution with the lysis buffer. Samples were then diluted to 10 ng/µL with loading buffer consisting of 125 mM Tris–HCl (pH 6.8), 1 mM EDTA, 20% glycerol, 5% SDS, 5% β-mercaptoethanol and bromophenol blue. Electrophoresis was performed using a Mini Protean III system (Bio-Rad Laboratories, Solna, Sweden). The separating gel consisted of 30% glycerol, 8% acrylamide, 0.16% bisacrylamide, 0.2 M Tris–HCl (pH 8.8), 0.1 M glycine, 0.4% SDS, 0.05% Temed and 1% ammonium persulfate. The stacking gel consisted of 30% glycerol, 4% acrylamide, 0.08% bisacrylamide, 70 mM Tris–HCl (pH 6.8), 4 mM EDTA, 0.4% SDS, 0.1% Temed and 1% ammonium persulfate. Protein lysates were heated at 95 °C for 10 min and 150 ng of protein was loaded into the gel. The gels were run at 4 °C during the whole migration period, including 40 min at 10 mA and 23 h 20 min at 140 V. The gels were silver-stained as described previously (Agbulut et al. 1996). The gels were scanned with an office scanner (CanoScan 9000F, Canon, The Netherlands, Amsterdam) and analysed with UN-SCAN-IT software (version 6.1, Silk Scientific Corporation, Orem, USA). The distribution of MHC isoforms was quantified using positive lane analysis and background correction (single region background value).

### RNA isolation, cDNA synthesis and quantitative polymerase chain reaction (qPCR)

Muscle samples (5–10 mg) were disrupted with a bead-homogenizer (TissueLyser LT, Qiagen) in 75 µL RA1 lysis buffer (Macherey-Nage, Dueren, Germany) containing 1% β-mercaptoethanol, and 250 µL TRIzol reagent (Life Technologies, Stockholm, Sweden). Tissue homogenate was then mixed with 100 µL chloroform (Sigma-Aldrich) before centrifugation. The aqueous phase (200 µL) was collected and mixed with an equal volume of chloroform. After centrifugation, 170 µL of the aqueous phase was precipitated with an equal volume of isopropanol at − 20 °C for 10 min. After centrifugation and an extra precipitation step with isopropanol, RNA was washed twice with 70% ethanol to remove excess salt. The pellet was dried at room temperature for 10 min, and dissolved in 12 µL nuclease-free water. The RNA samples were incubated 10 min at 55 °C and then kept on ice. Total RNA concentration and purity were assessed by measuring the optical density (230, 260 and 280 nm) with a spectrophotometer (Nanodrop 2000, Thermo Fisher Scientific). RNA samples were treated with genomic DNA removal kit (Heat and Run DNase, ArcticZymes, Tromsø, Norway) according to the manufacturer’s instructions, and RNA concentration was assessed again.

Total RNA (1 µg) was reversed-transcribed using iScript Select cDNA synthesis kit (Bio-Rad Laboratories). Total RNA was converted into cDNA using a final reaction volume of 20 µL containing: 2 µL of oligodT, 2 µL of random hexamers, 4 µL of 5X-iscript select reaction mix, 1 µL of reverse transcriptase and 9 µL of nuclease-free water. The reaction mix was incubated at 25 °C for 5 min, at 42 °C for 60 min, and at 85 °C for 5 min. After this, the samples were cooled down on ice, aliquoted, and stored at − 80 °C until further analysis. qPCR (final volume: 20 µL) was performed with samples loaded in duplicate using 5 µL of diluted cDNA (1/20 dilution from stock cDNA mixture), 10 µL of Rotor-Gene SYBR Green RT-PCR Master Mix (Qiagen), and 1 µL (Biorad Laboratories) or 0.4 µL (reverse and forward primers at 20 µM) of primers (list of primers presented in Table 1). The sequences of the forward and reverse primers for total *PGC1A, PGC1A* transcripts from exon 1a (*PGC1A-ex1a*) and truncated *PGC1A* were kindly provided by Jorge Ruas’s lab (Karolinska Institutet, Sweden) and were previously used (Ruas et al. 2012). *PGC1A-ex1a* primers can detect *PGC1A1* and *NT-PGC1A-a*, and truncated *PGC1A* primers can detect *PGC1A4*, *NT-PGC1A-a* and *NT-PGC1A-c* (not expressed in skeletal muscle) (Martinez-Redondo et al. 2015). *PGC1A1* isoform and *NT-PGC1A-a* are important regulators of mitochondrial biogenesis whereas *PGC1A4* isoform is induced after resistance exercise training and could promote muscle hypertrophy (Ruas et al. 2012; Martinez-Redondo et al. 2015). Several transcription factors associated with mitochondrial biogenesis and oxidative metabolism (Scarpulla et al. 2012), including nuclear respiratory factor 1 (NRF1), GA-binding protein transcription factor subunit alpha (GABPA, also called NRF2), mitochondrial transcription factor A (TFAM) and peroxisome proliferator-activated receptors alpha (PPARA) were also studied. The mRNA levels of three genes involved in redox homeostasis, a process closely connected to mitochondrial biogenesis (Ji et al. 2020), were assessed: Sirtuin 1 (*SIRT1*), nuclear factor erythroid 2-related factor 2 (*NFE2L2*) and superoxide dismutase 2 (*SOD2*). Finally, we determined the mRNA levels of two metabolic genes involved in substrate utilization (pyruvate dehydrogenase kinase 4, *PDK4*) and glucose transport (solute carrier family 2 member 4, *SLC2A4,* also called *GLUT4*)*.*

**Table 1:** List of primers used for qPCR analysis

Target	mRNA name	Catalogue number/sequence
*HPRT1*	Hypoxanthine phosphoribosyltransferase 1	qHsaCID0016375
*NFE2L2*	Nuclear factor (erythroid-derived 2)-like 2	qHsaCED0038543
*NRF1*	Nuclear respiratory factor 1	qHsaCID0007351
*PDK4*	Pyruvate dehydrogenase kinase, isozyme 4	qHsaCED0041886
*TFAM*	Transcription factor A, mitochondrial	qHsaCED0037846
*PPARA*	Peroxisome proliferator-activated receptor alpha	qHsaCID0011001
*GABPA*	GA-binding protein transcription factor, subunit alpha	qHsaCED0045648
*SIRT1*	Sirtuin 1	qHsaCED0042698
*SLC2A4*	Solute carrier family 2 member 4	qHsaCED0047488
*SOD2*	Superoxide dismutase 2, mitochondrial	qHsaCED0036418
Total *PGC1A*	Peroxisome proliferator-activated receptor gamma, coactivator 1 alpha	FW: 5′-CAGCCTCTTTGCCCAGATCTT-3' RV: 5′-TCACTGCACCACTTGAGTCCAC-3'
*PGC1A-ex1a*	Peroxisome proliferator-activated receptor gamma, coactivator 1 alpha (exon 1a)	FW: 5′-ATGGAGTGACATCGAGTGTGCT-3' RV: 5′-GAGTCCACCCAGAAAGCTGT-3'
Truncated *PGC1A*	Peroxisome proliferator-activated receptor gamma, coactivator 1 alpha (truncated forms)	FW: 5′-TCACACCAAACCCACAGAGA-3' RV: 5′-CTGGAAGATATGGCACAT-3'

*FW* forward primer, *RV* reverse primer

qPCR was performed using a Rotor Gene Q thermocycler (Qiagen) for 40 cycles (95 °C for 5 s and 60 °C for 30 s) followed by melting curve analysis. qPCR efficiency was estimated for each primer pair by performing standard curves obtained from serial dilutions of a pooled sample. We initially tested two reference genes: *RPLP0* (ribosomal protein lateral stalk subunit P0) and *HPRT1* (hypoxanthine phosphoribosyltransferase 1). *HPRT1* was selected and used for normalization because its expression was not affected by the experimental conditions (time and supplementation). The relative mRNA levels were calculated using the ΔΔ*C*_T_ method (Pfaffl 2001). The threshold cycle (*C*_T_) was calculated using the Rotor-Gene Q software (Qiagen), based on the qPCR conditions (auto-find threshold with slope correct adjustment) set up from the standard curves obtained for each gene. Since none of the target mRNAs were differently expressed between the two trials at baseline (pre-exercise), the mRNA levels were expressed as fold changes (FC) relative to pre-exercise values, which were set at 100%. This method is commonly used in studies with similar settings (i.e. cross-over design, post-exercise CHO restriction) (Pilegaard et al. 2005; Jensen et al. 2015; Mathai et al. 2008).

### Statistical analysis

**Fig. 2:**
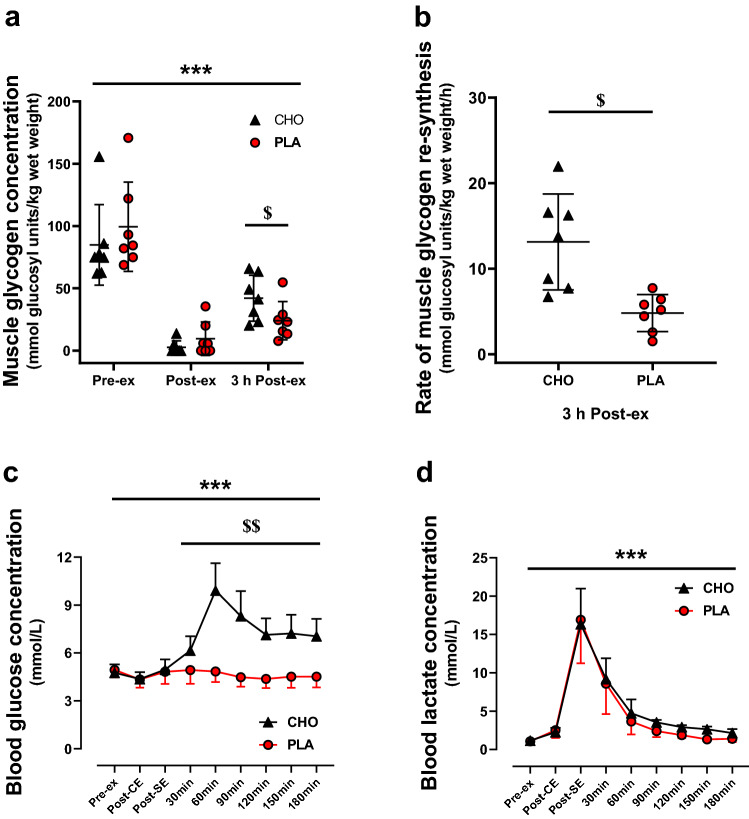
Muscle glycogen concentration (**a**), rate of muscle glycogen resynthesis (**b**), blood glucose concentration (**c**), and blood lactate concentration (**d**). Data are shown as means ± SD. Individual values are presented in **a** and **b**. ***P < 0.001: significant main effect of time. ^$^*P* < 0.05, ^$$^*P* < 0.01: significant differences between the CHO and PLA conditions. *Pre-ex* pre-exercise, *Post-CE* post-continuous cycling exercise, *Post-SE*, post-sprint cycling exercise. This figure was adapted from Cheng et al. (2020)

Data are presented as mean ± standard deviation (SD), and individual values are presented in Fig. 2a, b. All the statistical analyses were performed using GraphPad Prism (Graphpad Prism 8.0.2, San Diego, USA). As explained above, eight participants were included in the analysis of this study. Power output of the SIE was analyzed from only seven subjects because it was not recorded during the SIE of the PLA trial for one subject. In addition, one subject was excluded from the analysis of muscle glycogen (Fig. 2a, b) due to outlier values (i.e. > 2 SD) above the mean at post-exercise for the CHO trial. Similarly, one subject was excluded from the analysis of *PDK4* mRNA (Fig. 6a) due to outlier values at the time points post-exercise and 3 h post-exercise for the CHO trial.

Shapiro–Wilk tests were used to check normality before selecting the appropriate parametric or non-parametric statistical tests. Paired *t* tests were used to analyze the mean power, mean HR_,_ peak HR, mean RPE and the rate of muscle glycogen resynthesis for both conditions. For the analysis of blood glucose concentration, and for the analysis of the mRNA levels of total *PGC1A, TFAM, SIRT1* and *GABPA*, two-way repeated-measures analysis of variance (2-way RM ANOVA) tests were used to assess the effect of time, supplementation (CHO vs PLA), and time × supplementation interaction*.* When an interaction was observed, a Sidak multiple comparisons test was used to compare the PLA and CHO conditions. For the analysis of blood lactate and muscle glycogen concentration, as well as for the analysis of the mRNA levels of *PGC1A-ex1a*, truncated *PGC1A, NFE2L2, NRF1, PDK4, SOD2, PPARA, SLC2A4,* Friedman tests were used to assess the effect of time in both experimental trials. Then, Wilcoxon’s matched-pairs signed-rank tests with Bonferroni corrections were performed to compare the conditions (CHO vs. PLA) at each time point. The level of significance was set at *P* < 0.05.

## Results

### Strenuous glycogen-depleting cycling session

Participants performed the 60-min CCE at a power eliciting 60% *V*O_2max_ (2.11 ± 0.40 W/kg body mass) in both the CHO and PLA experimental trials. During this exercise, mean RPE (15.3 ± 0.7 and 15.0 ± 1.1, respectively) and mean HR (150 ± 9 bpm and 147 ± 11 bpm, respectively) were similar in both the CHO and PLA conditions. During the SIE, the mean power output was similar in both the CHO and PLA trials (7.03 ± 0.68 W/kg body mass and 6.86 ± 0.55 W/kg body mass, respectively), while the average of the peak HR of each cycling sprint was slightly higher (2%) for the CHO trial compared to the PLA trial (168 ± 10 bpm and 164 ± 11 bpm, respectively; *P* < 0.05).

Before exercise, muscle glycogen concentration was not significantly different between the two randomized trials (CHO trial: 84.9 ± 32.3 mmol glucosyl units/kg wet weight; PLA trial: 99.4 ± 35.8 mmol glucosyl units/kg wet weight; *P* = 0.12) (Fig. 2a). After exercise, muscle glycogen concentration was severely decreased, reaching 2.7 ± 5.2 mmol glucosyl units/kg wet weight in the CHO condition and 9.6 ± 13.4 mmol glucosyl units/kg wet weight in the PLA condition. At 3 h post-exercise, muscle glycogen concentration was significantly higher in the CHO trial than in the PLA trial (42.1 ± 18.4 mmol glucosyl units/kg wet weight and 24.0 ± 15.4 mmol glucosyl units/kg wet weight, respectively; *P* < 0.05). The rate of muscle glycogen resynthesis during the 3-h recovery period was significantly higher in the CHO trial than the PLA trial (13.1 ± 5.6 and 4.8 ± 2.2 mmol glucosyl units/kg wet weight/h, respectively; Fig. 2b, *P* < 0.05), demonstrating that CHO ingestion promoted muscle glycogen resynthesis. In addition, blood glucose concentration, which was similar in both the CHO and PLA trials before exercise (4.76 ± 0.52 mM and 4.94 ± 0.47 mM, respectively), markedly increased in response to CHO ingestion only (time × supplementation interaction, *P* < 0.001), reaching the highest values at 60 min post-exercise (CHO trial: 9.91 ± 1.70 mM; PLA trial: 4.84 ± 0.66 mM; *P* < 0.01) (Fig. 2c). Blood glucose concentration was significantly higher in the CHO condition than in the PLA condition from 30 min post exercise (*P* < 0.01). Blood lactate concentration, which was similar in both the CHO and PLA experimental trials before exercise (1.16 ± 0.24 mM and 1.06 ± 0.27 mM, respectively), markedly increased after the SIE, reaching 16.35 ± 4.63 mM and 16.91 ± 5.66 mM in the CHO and PLA conditions, respectively (main effect of time, *P* < 0.001) (Fig. 2d). Blood lactate concentration progressively decreased to 2.15 ± 0.52 mM and 1.40 ± 0.43 mM 180 min post exercise in the CHO and PLA conditions, respectively. There were no significant differences in blood lactate concentration between the two trials at any time points.

### mRNA levels of genes associated with mitochondrial biogenesis and oxidative metabolism

**Fig. 3:**
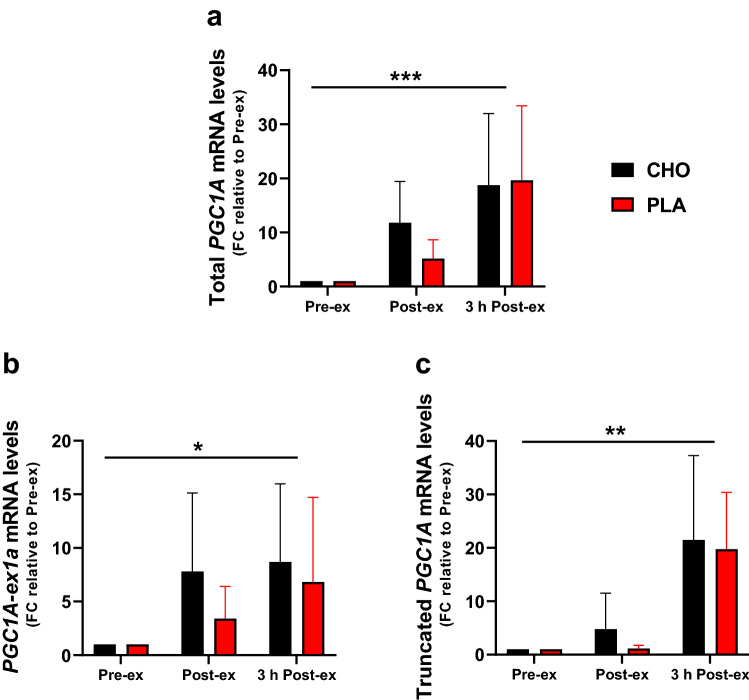
mRNA levels of total *PGC1A* (**a**), *PGC1A-ex1a* (**b**), and truncated *PGC1A* (**c**). The results values are expressed as fold change (FC) relative to the pre-exercise values. Data are shown as means ± SD. *P < 0.05, **P < 0.01, ***P < 0.001: significant main effect of time

The mRNA levels of total *PGC1A* were highly increased after exercise (time effect: *P* < 0.001), resulting in a five to tenfold increase directly after exercise and a ~ 20-fold increase 3 h after exercise (Fig. 3a). *PGC1A-ex1a* mRNA increased 3.5- to 8-fold directly after exercise and 7- to 8.5-fold 3 h post exercise (time effect: *P* < 0.05) (Fig. 3b). A marked increase in truncated *PGC1A* mRNA levels (~ 20-fold) was observed 3 h post-exercise in both trials (time effect: *P* < 0.01) (Fig. 3c). CHO restriction in the PLA trial during the three hours following exercise did not affect the mRNA levels of total *PGC1A*, *PGC1A-ex1a* and truncated *PGC1A*.

**Fig. 4:**
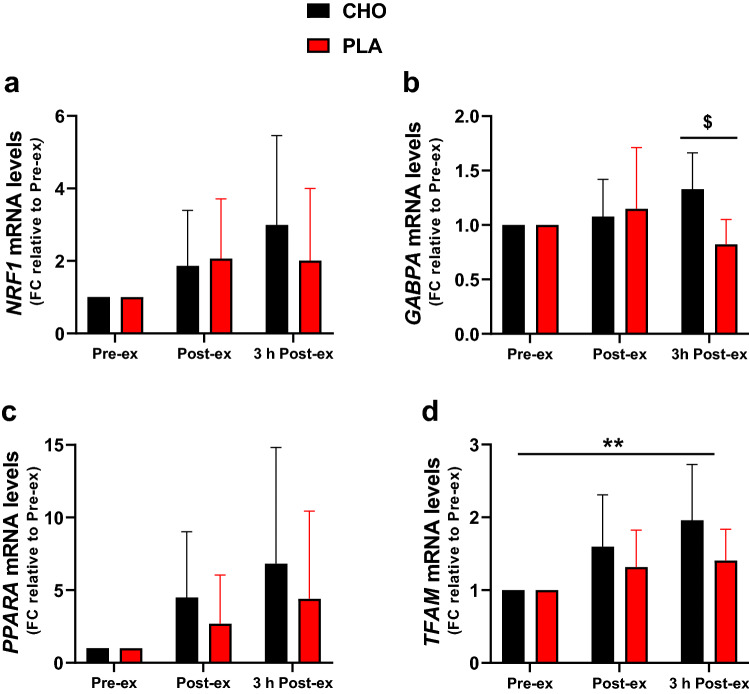
mRNA levels of transcription factors closely related to *PGC1A*. mRNA levels of *NRF1* (**a**), *GABPA* (**b**), *PPARA* (**c**), and *TFAM* (**d**). The results values are expressed as fold change (FC) relative to the pre-exercise values. Data are shown as means ± SD. **P < 0.01: significant main effect of time. ^$^*P* < 0.05, significant differences between the CHO and PLA conditions

The mRNA levels of *NRF1* (Fig. 4a), *GABPA* (Fig. 4b), and *PPARA* (Fig. 4c) were not significantly affected by time, while *TFAM* mRNA levels (Fig. 4d) slightly increased after exercise (~ 1.5- to 2-fold; time effect: *P* < 0.01). CHO restriction during the 3-h post-exercise period did not affect the abundance of *NRF1, TFAM,* and *PPARA*, while *GABPA* mRNA levels were slightly lower in the PLA than the CHO conditions at this time point (*P* < 0.05).

**Fig. 5:**
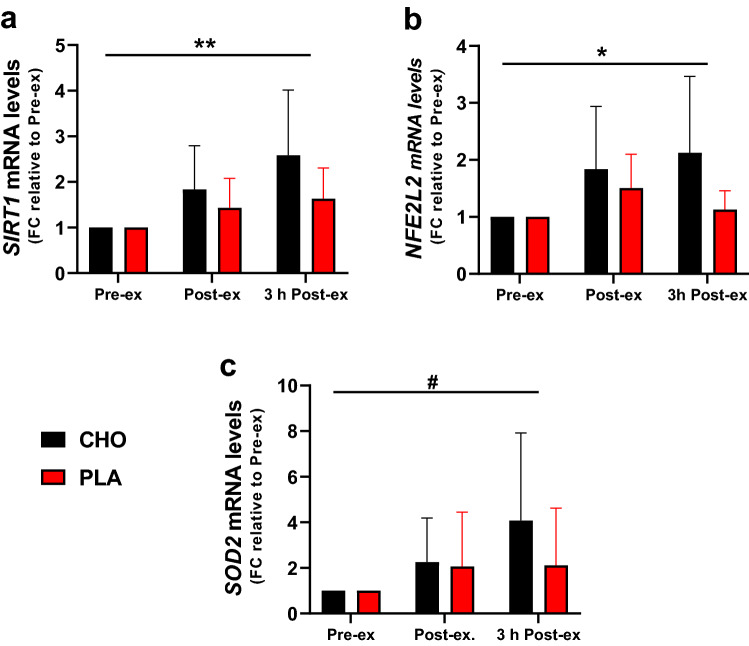
mRNA levels of genes regulating redox homeostasis. mRNA levels of *SIRT1* (**a**), *NFE2L2* (**b**) and *SOD2* (**c**). The results values are expressed as fold change (FC) relative to the pre-exercise values. Data are shown as means ± SD. *P < 0.05, **P < 0.01: significant main effect of time. ^#^*P* < 0.05: significant main effect of time in the CHO condition only

The mRNA levels of *SIRT1* (Fig. 5a) and *NFE2L2* (Fig. 5b) were significantly increased during the recovery period (time effect: *P* < 0.01 and *P* < 0.05, respectively), while this result was only observed for *SOD2* in the CHO trial (time effect: *P* < 0.05; Fig. 5c). CHO restriction during the three hours following exercise did not affect the mRNA abundance of *SIRT1*, *NFE2L2* and *SOD2*.

**Fig. 6:**
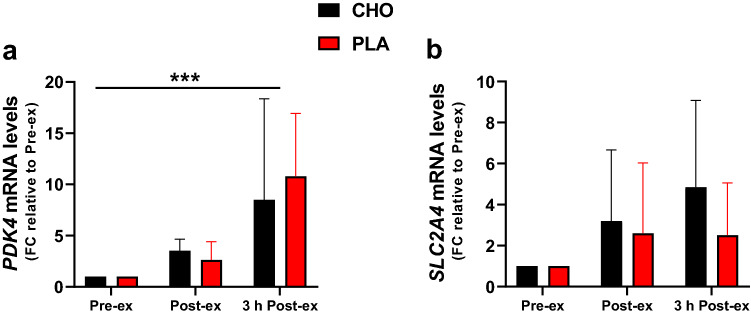
mRNA levels of genes associated with substrate utilization and glucose transport. mRNA levels of *PDK4* (**a**) and *SLC2A4* (**b**). The results values are expressed as fold change (FC) relative to the pre-exercise values. Data are shown as means ± SD. ***P < 0.001: significant main effect of time

*PDK4* mRNA levels were increased ~ threefold directly after exercise and ~ tenfold 3 h post exercise (time effect: *P* < 0.001; Fig. 6a). *SLC2A4* mRNA levels were not significantly affected by time (Fig. 6b). 3 h post exercise, the mRNA levels of these genes were not influenced by CHO restriction.

## Discussion

Reduced CHO availability has been proposed to be a potent metabolic regulator of intracellular signaling pathways promoting endurance-training adaptations in skeletal muscle (e.g., mitochondrial biogenesis, substrate utilization, oxidative metabolism) (Hearris et al. 2018; Mata et al. 2019). In the current study, we tested the hypothesis that CHO restriction following a severely glycogen-depleting and highly metabolically-demanding exercise would potentiate the acute molecular response associated with stimulation of mitochondrial biogenesis and oxidative metabolism adaptation in human skeletal muscle. Our results indicated that post-exercise CHO restriction following a strenuous cycling session that combined CCE and SIE did not amplify the mRNA levels of genes associated with muscle adaptation to endurance exercise.

In this study, we adopted an experimental exercise session that induced both severe muscle glycogen depletion and a high metabolic stress. Indeed, this exercise session led to more than 90% muscle glycogen depletion while blood lactate concentration was highly elevated after the last sprint. In addition, the mRNA levels of *PGC1A*, a central regulator of mitochondrial biogenesis and oxidative metabolism (Correia et al. 2015), were substantially increased 3 h post exercise (~ 20-fold). Intriguingly, the magnitude of the changes in *PGC1A* observed in the current study is much higher than that observed in previous studies which consisted of solely moderate-intensity exercise (Jensen et al. 2015; Pilegaard et al. 2005; Mathai et al. 2008), or high intensity interval exercise (HIIE) (Bartlett et al. 2013) (Perry et al. 2010), or SIE (Cochran et al. 2014). In our present study, these pronounced changes in mRNA levels are most likely the result of the combination of severe muscle glycogen depletion and metabolic stress caused by this strenuous exercise session (i.e. CCE following by SIE), although other factors could have also played a role (e.g. the time points of muscle sample collection, the training status of the participants, the form of activity, etc.). For instance, it has been reported that transient and large increases in *PGC1A* mRNA levels occur after HIIE, but the mRNA response to exercise was attenuated after several sessions as the muscle adapted to the exercise challenge (Perry et al. 2010).

Interestingly, the abundance of both *PGC1A-ex1a* and truncated *PGC1A* mRNA highly increased during the recovery period, but the changes observed 3 h after exercise for truncated *PGC1A* were of higher magnitude than those observed for *PGC1A-ex1a*. Since *PGC1A-ex1a* primers and truncated *PGC1A* primers can both detect *NT-PGC1A-a* (see in the method section), the large increased expression of truncated *PGC1A* observed at 3 h post exercise is most likely the result of *PGC1A4* up-regulation. *PGC1A1* isoform (and *NT-PGC1A-a*) specifically promotes mitochondrial biogenesis in response to endurance exercise, whereas overexpression of *PGC1A4* isoform promotes muscle hypertrophy in mice and is induced in humans after resistance training (Ruas et al. 2012; Martinez-Redondo et al. 2015). However, some human studies have shown that the mRNA levels of truncated *PGC1A* isoforms, including *PGC1A4*, are highly increased in the early period (2–6 h) following endurance exercise, and are not preferentially increased in response to resistance exercise (Ydfors et al. 2013; Lundberg et al. 2014). Since a 6-week cycling SIE training intervention does not increase muscle fiber size (Joanisse et al. 2015), it seems that the role of *PGC1A4* is not solely to induce muscle hypertrophy but could also be to promote endurance-training adaptations, such as angiogenesis (Ruas et al. 2012; Thom et al. 2014).

The main finding of this study is that CHO restriction following a severely glycogen-depleting and highly metabolically-demanding exercise does not increase the mRNA levels of numerous factors involved in mitochondrial biogenesis and oxidative metabolism in human skeletal muscle. From the different categories of transcripts studied (*PGC1A*, transcription factors closely related to *PGC1A*, regulators of redox homeostasis, and factors associated with substrate utilization and glucose transport), only *GABPA* mRNA levels were slightly lower in the PLA trial compared to the CHO trial 3 h post exercise. This result remains unclear, but the small differences observed may not be of biological relevance. In the current study, pre-exercise muscle glycogen stores were in the same range as those observed in a rested and fasted state in a similar population (i.e., physically active males) (Blom et al. 1987; Maehlum et al. 1977). The fasted state and training status (i.e., recreationally active) of the subjects, the absence of CHO supplementation during exercise and the exhausting characteristic of our exercise protocol most likely explain why muscle glycogen depletion was so substantial in our current study. In addition, *PDK4,* which is highly sensitive to low CHO availability, was shown to be upregulated in response to CHO restriction (Hammond et al. 2019; Cluberton et al. 2005). Here, *PDK4* mRNA was substantially expressed after exercise, especially 3 h post exercise (~ tenfold), but no differences were found between the CHO and PLA trials. Our findings suggest that post-exercise CHO restriction did not further enhance mRNA levels of this metabolic gene because the stimuli associated with our exercise protocol (i.e. muscle severe glycogen depletion and high metabolic stress) were sufficient to maximize the expression of *PDK4*.

The muscle glycogen threshold hypothesis, whereby a low level of muscle glycogen during exercise (100–300 mmol glucosyl units/kg dry muscle weight) is especially potent in modulating the activation/expression of key molecular regulators of endurance-training adaptations has been recently proposed (Impey et al. 2018). In our study, muscle glycogen concentration was very low directly after exercise, and remained within the frame of 100–300 mmol glucosyl units/kg dry muscle weight after the short recovery period (3 h) in all participants of both the PLA and CHO trials [average of ~ 105 and 180 mmol glucosyl units/kg dry muscle weight, respectively; data converted from wet-weight values to dry-wet values using the conversion factor 4.325 (Murray and Rosenbloom 2018)]. These results may explain the absence of differential molecular response at 3-h post exercise between the two trials.

Reactive oxygen and nitrogen species (RONS) have been recently proposed to be a primary stimulus of mitochondrial biogenesis in skeletal muscle (Merry and Ristow 2016). RONS production highly increases in response to intense muscle contraction and intense exercise, at least in mice (Henríquez-Olguín et al. 2019; Place et al. 2015). Even though we did not directly measure RONS production after cycling sprints, our results indicate that the expression of the two redox sensitive genes SIRT1 and NFE2L2 was increased during the recovery period following exercise, but was not affected by post-exercise CHO restriction. Thus, it is likely that post-exercise CHO restriction does not enhance or suppress RONS-stimulated pathways involved in mitochondrial biogenesis.

### Study limitations

Due to the difficulty of recruiting elite endurance athletes, this study was performed with recreationally active participants. Future experiments would be helpful to confirm our results in elite endurance athletes, since training status influences glycogen stores, as well as hormonal and metabolic changes in response to exercise (Hearris et al. 2018). In addition, collecting additional muscle samples at later time point during the recovery period (i.e. 5–8 h) would have been relevant to assess the expression of *PGC1A* when it potentially peaks (Pilegaard et al. 2005). This was not realized because our primary objective was to investigate the putative benefits of short post-exercise CHO restriction. Pre-exercise muscle glycogen concentration was slightly but not significantly lower in the CHO trial than the PLA trial. This slight difference might be explained by several parameters, including the absence of standardized meal on the evening before the exercise session, and the physical activity performed during the days preceding the experiments (although the participants were asked to refrain from any strenuous exercise during the last 3 days prior to each experimental session). Whether these limitations would have affected the results of our study is uncertain. Another limitation concerns the fact that changes in mRNA levels may not always reflect eventual changes at the translational level. Unfortunately, the assessment of protein levels was not possible due to the small size of the biopsy samples collected. Finally, a rather large variability was observed for the biological parameters analyzed in this study. Although including more participants may have increased the power of our analysis, when taken into context, our results are consistent with the majority of previous studies that showed no effect of CHO restriction following glycogen-depleting exercise on the acute mRNA levels of genes involved in mitochondrial biogenesis and muscle metabolism (Pilegaard et al. 2005; Jensen et al. 2015; Mathai et al. 2008).

## Conclusion

We conclude that CHO restriction after a glycogen-depleting and metabolically-demanding cycling session does not increase the acute mRNA levels of genes involved in mitochondrial biogenesis and oxidative metabolism in human skeletal muscle. Our results indicate that the early molecular response to exercise was highly stimulated by the stimuli associated with our protocol (i.e. glycogen-depleting and metabolically-demanding exercise, fasted state, putative RONS production, plus the absence of CHO supplementation during exercise). Thus, the additional metabolic stress (i.e. post-exercise CHO restriction) had no additional benefit in amplifying the early gene response to exercise.

## Abbreviations

CCE: Continuous cycling exercise
CHO: Carbohydrate
GABPA: GA-binding protein transcription factor, subunit alpha
HPRT1: Hypoxanthine phosphoribosyltransferase 1
HIIE: High-intensity interval exercise
HR: Heart rate
MHC: Myosin heavy chain
NFE2L2: Nuclear factor (erythroid-derived 2)-like 2
NRF1: Nuclear respiratory factor 1
PDK4: Pyruvate dehydrogenase kinase, isozyme 4
PGC1α: Peroxisome proliferator-activated γ-receptor coactivator 1alpha
PLA: Placebo
PPARA: Peroxisome proliferator-activated receptor alpha
SIE: Sprint interval exercise
SIRT1: Sirtuin 1
SLC2A4: Solute carrier family 2 member 4
SOD2: Superoxide dismutase 2, mitochondrial
TFAM: Transcription factor A, mitochondrial
